# Construction of New Ligation-Independent Cloning Vectors for the Expression and Purification of Recombinant Proteins in Silkworms Using BmNPV Bacmid System

**DOI:** 10.1371/journal.pone.0064007

**Published:** 2013-05-10

**Authors:** Tatsuya Kato, James R. Thompson, Enoch Y. Park

**Affiliations:** 1 Laboratory of Biotechnology, Department of Applied Biological Chemistry, Faculty of Agriculture, Shizuoka University, Suruga-ku, Shizuoka, Japan; 2 Department of Physiology and Biomedical Engineering, Mayo Clinic College of Medicine, Rochester, Minnesota, United States of America; 3 Laboratory of Biotechnology, Integrated Bioscience Section, Graduate School of Science and Technology, Shizuoka University, Suruga-ku, Shizuoka, Japan; 4 Laboratory of Biotechnology, Research Institute of Green Science and Technology, Shizuoka University, Suruga-ku, Shizuoka, Japan; Natural Resources Canada, Canada

## Abstract

A ligation independent cloning (LIC) system has been developed to facilitate the rapid and high-efficiency cloning of genes in a *Bombyx mori* expression system. This system was confirmed by the expression of human microsomal triglyceride transfer protein (hMTP) fused with EGFP in silkworm larvae and pupae. Moreover, hMTP and human protein disulfide isomerase (hPDI) genes were inserted into two LIC vectors harboring gcLINK sequences and were combined by using the LIC through gcLINK sequences. The constructed vector was incorporated into the *Bombyx mori* nucleopolyhedrovirus (BmNPV) bacmid, and injected into silkworm larvae. The expressed hMTP-hPDI complex was purified from the fat bodies of silkworm larvae. This LIC vector system was applied to express the E1, E2, and E3 subunits of human α-ketoglutarate dehydrogenase (KGDH) in silkworm larvae. The expressed proteins were purified easily from fat bodies using three different affinity chromatography steps. The LIC vectors constructed as described in this report allow for the rapid expression and purification of recombinant proteins or their complexes by using the BmNPV bacmid system.

## Introduction

Generally, to produce recombinant proteins, target genes are amplified by PCR with primers containing restriction enzyme sites. PCR products and expression vectors are digested by restriction enzymes and ligated using T4 DNA ligase. Using this protocol, the available restriction enzymes depend on the nucleotide sequences of target genes, and different enzymes should be used when many target genes are cloned in parallel.

Ligation-independent cloning (LIC) has been developed as a new cloning method, which eliminates the use of restriction endonuclease digestion and ligation of PCR products [1], [2]. LIC circumvents the limitations of traditional gene cloning methods because any PCR products can be inserted into LIC-compatible cloning vectors without restriction enzymes and T4 DNA ligase. In LIC, PCR primers are designed to have LIC-compatible 5′ extension sequences. PCR products and vectors are treated with T4 DNA polymerase in the presence of a single deoxyribonucleotide triphosphate, which generates specific 12–20 nucleotide single stranded overhangs. The PCR products are then annealed with vectors which have complementary overhangs, and transformation into *Escherichia coli* is performed. Host repair enzymes yield circular plasmids.

LIC is now used for the expression of fusion protein [3], [4], co-expression of proteins [5], and high-throughput expression and purification of proteins [6], [7]. LIC is especially suitable for high-throughput expression of recombinant proteins [6], [7] and automation of gene expression [8], because LIC allows for very high cloning efficiency with a minimal background. However, LIC has been mainly used in the *E. coli* expression system, although there are a few reports of using LIC in other expression systems [9], [10]. This LIC system is similar to the In-Fusion HD technique (developed by Clontech Laboratories Inc., CA. USA), but the latter requires expensive In-Fusion HD enzyme for gene cloning. In contrast, only conventional enzymes, restriction enzymes, and T4 DNA polymerase are used for gene cloning in the LIC system. The efficiency of gene cloning using this LIC system is higher than that using conventional ligation, and comparable to the In-Fusion technique.

In this study, LIC vectors that can express affinity tag-fused recombinant proteins in the N- or C-terminal were constructed for high-throughput expression and purification of recombinant proteins in a silkworm expression system. Expression and purification of human α-ketoglutarate dehydrogenase (KGDH) subunits (E1, E2, E3) in silkworm larvae showed the feasibility of using LIC vectors. Moreover, an improved LIC vector, allowing the fusion of two expression plasmids into one was also constructed for expression of a protein complex based on the ‘LINK’ sequence [11], [12]. A newly created ‘LINK’ sequence was incorporated into the improved LIC vector and used for successful co-expression of human microsomal triglyceride transfer protein (hMTP) and human protein disulfide isomerase (hPDI) protein complex.

## Materials and Methods

### Construction of LIC vectors for the expression of recombinant proteins fused with affinity tag C-terminally

**Figure 1:**
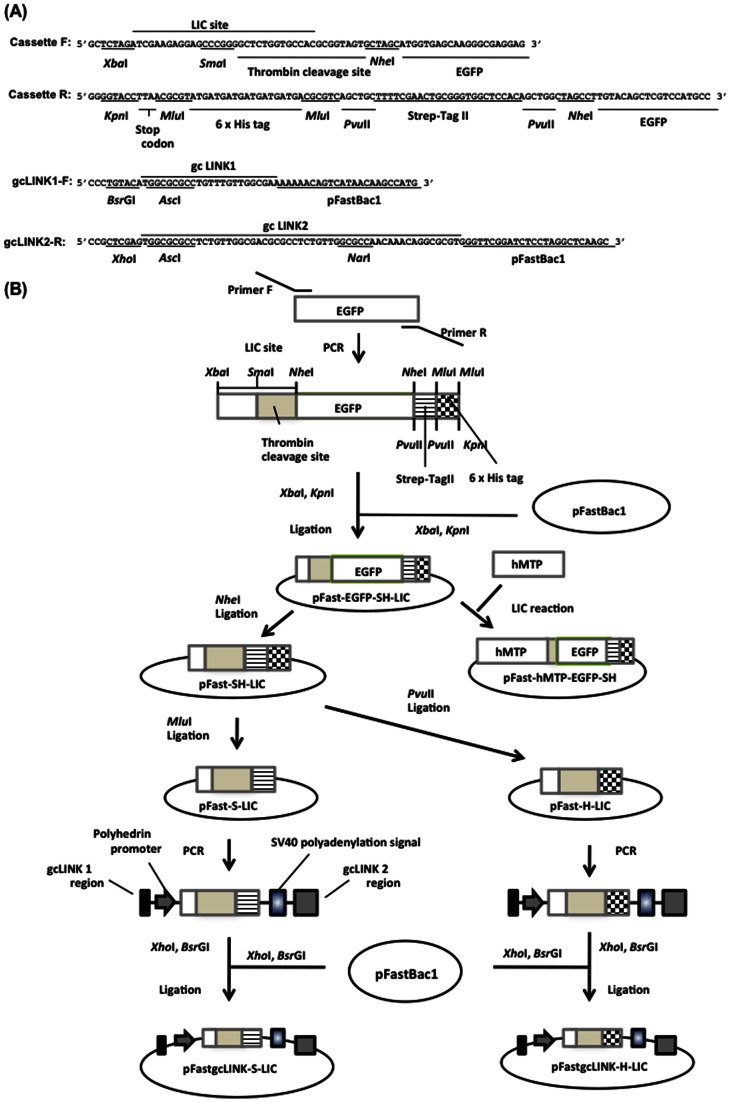
Sequences of primers used for construction of pFastBac-EGFP-SH-LIC, pFastgcLINK-S-LIC, and pFastgcLINK-H-LIC and scheme for construction of various LIC vectors for C-terminally tagged protein expression in silkworms. (A) EGFP-fusion protein expression cassette was amplified by using Cassette F and Cassette R. Cassette F contained the LIC site and thrombin cleavage site. Cassette R contained Strep-tag II and 6× histidine tag sequences. gcLINK1-F and gcLINK2-R were used to insert each gcLINK region into LIC vector. gcLINK1-F and gcLINK2-R contained gcLINK1 and gcLINK2 regions, respectively. (B) Constructed pFastgcLINK-S-LIC and pFastgcLINK-H-LIC were used for the co-expression of hMTP-S and hPDI-H, as shown in Figure 2.

**Figure 2:**
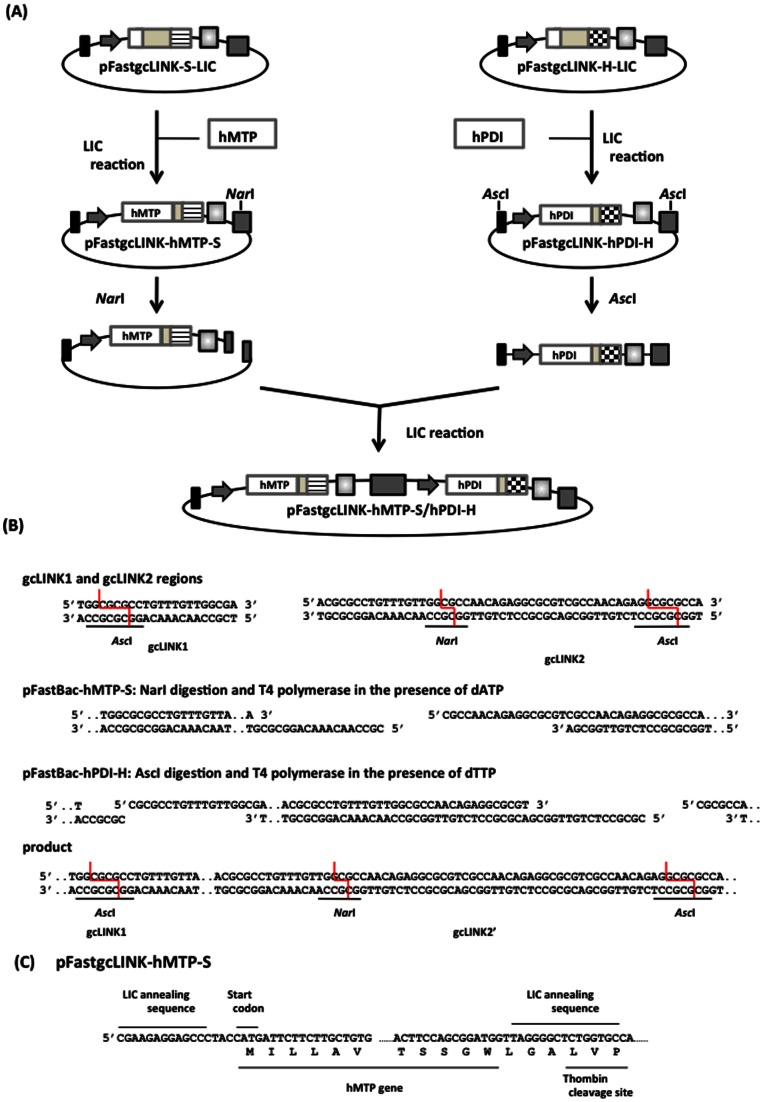
Scheme for construction of hMTP-hPDI co-expression vector (pFastgcLINK-hMTP-S/hPDI-H) based on pFastgcLINK-S-LIC, pFastgcLINK-H-LIC, and gcLINK sequences, their restriction enzyme digestion and annealing sites. (A) hMTP and hPDI were inserted into pFastgcLINK-S-LIC and pFastgcLINK-H-LIC, respectively. Constructed vectors (pFastgcLINK-hMTP-S and pFastgcLINK-hPDI-H) were combined with each other by LIC. Constructed vector was named pFastgcLINK-hMTP-S/hPDI-H. (B) gcLINK1 and gcLINK2 regions were digested by *Asc* I and *Nar* I, respectively, and treated with T4 DNA polymerase in the presence of dATP or dTTP. Each 5′- overhang can be annealed and each vector can be combined into one. (C) Sequence of hMTP gene in the pFastgcLINK-hMTP-S vector is shown. hMTP gene was successfully inserted into LIC site in this vector. Sequence of the hPDI gene in pFastgcLINK-hPDI-H vector is not shown, but the hPDI gene was also successfully inserted in-frame into the vector.

Plasmid construction is illustrated in Figures 1 and 2. To construct various LIC vectors, LIC-specific forward (Cassette F) and reverse primers (Cassette R) were designed as shown in Figure 1A. Cassette F contains the LIC sequence, the thrombin cleavage site, and the 5′-sequence of the EGFP gene. Cassette R contains the 6× histidine tag, Strep-tag II sequences, and 3′-sequence of the EGFP gene. PCR was performed using these primers, and the EGFP gene was used as a template. The PCR product treated with *Xba* I and *Kpn* I was inserted into the *Xba* I–*Kpn* I site of pFastBac 1 (Invitrogen, San Diego, CA, USA). The constructed plasmid was named pFastEGFP-SH-LIC (Figure 1B). EGFP was removed from pFastEGFP-SH-LIC by *Nhe* I and self-ligation, and pFast-SH-LIC was produced. Digestion of pFast-SH-LIC by *Mlu* I and *Pvu* II, respectively, and self-ligation, produced pFast-S-LIC and pFast-H-LIC.

Two modified LINK sequences allowing the joining of plasmids in tandem were also designed [12]. gcLINK1 and gcLINK2 include the GC rich recognition sites for the restriction enzyme *Asc* I (gcLINK1) or *Asc* I and *Nar* I (gcLINK2) (Figure 2B). The construction of tandem plasmids allows the co-expression of two subunits of a protein complex [11], [12]. Two expression cassettes can be in tandem connected in one vector through gcLINK1 and 2 sequences. gcLINK-specific primers (gcLINK1-F, gcLINK2-R) (Figure 1A) were used to insert gcLINK sequences into pFast-S-LIC and pFast-H-LIC. gcLINK1-F and gcLINK2-R primers have gcLINK1 and gcLINK2 sequences, respectively. The region containing the polyhedrin promoter, LIC sequence, and the SV40 polyadenylation signal region was amplified using these primers and pFast-S-LIC or pFast-H-LIC as a template. Each amplified fragment treated by *Bsr* GI and *Xho* I was inserted into the *Bsr* GI–*Xho* I site of pFastBac1. Thus, pFastgcLINK-S-LIC and pFastgcLINK-H-LIC were constructed (Figure 1B).

### LIC reaction

Plasmid construction by LIC reaction is also described in Figure 2. pFast-S-LIC and pFast-H-LIC were treated with *Sma* I and incubated at 65°C for 20 min to inactivate enzyme activity. T4 DNA polymerase (2.5 U) (Fermentas INC, Glen Burnie, MD, USA), which has both activities of 5′-3′ polymerase and 3′-5′ exonuclease, and 2.5 mM dTTP, were added into reaction mixtures and incubated at 25°C for 30 min, followed by heating at 65°C for 20 min to inactivate T4 DNA polymerase. Because of the 3′-5′ activity of polymerase the nucleotides (“A”, “G” and “C”) are removed from 3′-ends until the first “T” residue is reached. When T4 DNA polymerase comes to this first “T”, its polymerase reaction dominates and its 3′-5′ exonuclease reaction stops. This reaction leads two specific 5′-overhangs in this LIC vector of 10 and 12 bases, respectively.

hMTP and hPDI genes were amplified by PCR using Human MTP F (5′ CGAAGAGGAGCCCTACCATGATTCTTCTTGCTGTG 3′), Human MTP R (5′ GGCACCAGAGCCCCTAACCATCCGCTGGAAGT 3′), Human PDI F (5′ CGAAGAGGAGCCCTACCATGCTGCGCCGCGCTCTG 3′), and Human PDI R (5′ GGCACCAGAGCCCCTACAGCTTTCTGATCATC 3′) primer sets, respectively. The ER retention sequence (last 4 amino acids of hPDI) was removed from the intact hPDI sequence. Amplified genes were purified and treated with T4 DNA polymerase according to the protocol described above. Then, T4 DNA polymerase-treated hMTP and hPDI were mixed with T4 DNA polymerase-treated pFastgcLINK-S-LIC and pFastgcLINK-H-LIC, respectively, and heated at 65°C for 5 min. The mixtures were then cooled to room temperature for 1 h to anneal each other. Two microliters of 25 mM EDTA was added to each mixture before *E. coli* DH10B (Invitrogen) transformation. The constructed plasmids were named pFastgcLINK-hMTP-S and pFastgcLINK-hPDI-H. Applying the same protocol described above, pFastBac-hMTP-EGFP-SH was constructed using pFastEGFP-SH-LIC and hMTP genes amplified by PCR.

To connect pFastgcLINK-hMTP-S with pFastgcLINK-hPDI-H, pFastgcLINK-hMTP-S and pFastgcLINK-hPDI-H were digested by *Nar* I and *Asc* I, respectively, and each digested plasmid was treated with T4 DNA polymerase in the presence of 2.5 mM dATP or dTTP. *Nar* I-Digested pFastgcLINK-hMTP-S and an ∼ 2.8 kbp *Asc* I-digested fragment including the hPDI gene were purified. Overhang sequences of LINK1 and 2 regions after T4 DNA polymerase treatment are shown in Figure 2B. Each purified fragment was mixed, heated, and then cooled at room temperature to anneal each other at the complementary sequences. Two microliters of 25 mM EDTA was added to each mixture before *E. coli* DH5α transformation. The constructed plasmids were designated as pFastgcLINK-hMTP-S/hPDI-H (Figure 2).

### Construction of LIC vectors for the expression of recombinant proteins fused with an affinity tag in N-terminal

**Figure 3:**
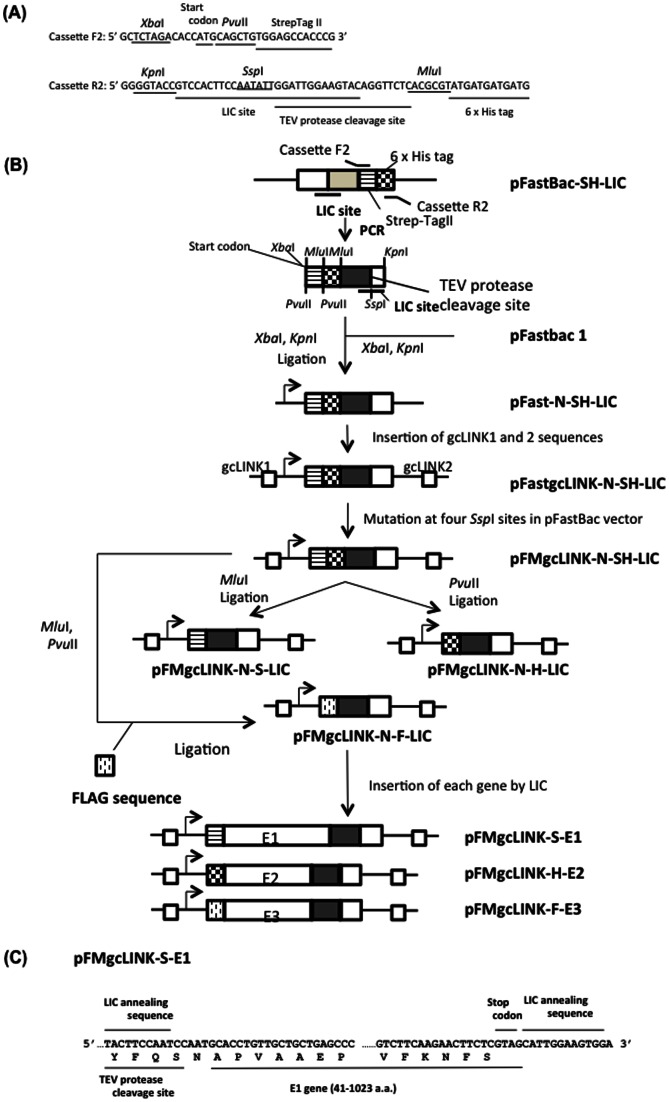
Sequences of primers used for amplification of LIC cassette containing start codon, Strep-tag II sequence, 6× histidine tag sequence, TEV protease cleavage site sequence, and LIC site for N-terminal tagged protein expression, and scheme for LIC construction vectors. (A) LIC cassette for N-terminal tagged protein expression was amplified using primer F2 and primer R2. Amplified LIC cassette has start codon, Strep-tag II sequence, 6× histidine tag sequence, TEV protease cleavage site sequence, and *Ssp* I site in the LIC site. (B) pFMgcLINK-S-E1, pFMgcLINK-H-E2, and pFMgcLINK-F-E3 were constructed for each KGDH subunit expression in silkworms. (C) The sequence of E1 gene in pFMgcLINK-S-E1 vector is shown. E1 gene was successfully inserted into the LIC site in-frame in this vector. The sequences of E2 and E3 genes in constructed vectors are not shown, but these genes were also successfully inserted in-frame into the vector.

A LIC cassette for the expression of recombinant proteins fused with an affinity tag in the N-terminal was amplified by PCR using primers F2 and R2, and pFast-SH-LIC, which was constructed in this study as a template (Figure 3). An amplified fragment containing the Start codon, StrepTag II sequence, 6× histidine tag sequence, TEV protease cleavage site, and LIC site (*Ssp* I) was ligated into the *Xba* I – *Kpn* I site in pFastbac 1. gcLINK1 and gcLINK2 sequences were inserted into the resulting plasmid (pFast-N-SH-LIC) by the same manner as described in “Construction of LIC vectors for the expression of recombinant proteins fused with affinity tag C-terminally” in Materials and Methods. The resulting plasmid, pFastgcLINK-N-SH-LIC, had 5 *Ssp* I sites. Four of the *Ssp* I sites in this vector, except for that in the LIC sequence, were mutated by using the Quikchange Lightening Multi site-Directed Mutagenesis Kit (Agilent Technologies, Inc., Santa Clara, CA, USA) using the following primers (pF-ssp441: 5′ CGAATTTTAACAAAAGATTAACGTTTACAAT 3′, pF-ssp572: 5′ GATAAATGCTTCAATAAGATTGAAAAAGGAAG 3′, pF-3896: 5′ CGTATACTCCGGAAGATTAATAGATCATGG 3′, pF-3996: 5′ TAAAAAAACCTATAAAGATTCCGGATTATTC 3′). The mutated plasmid (pFMgcLINK-N-SH-LIC) was digested by *Mlu* I or *Pvu* II, and each digested plasmid was self-ligated, resulting in pFMgcLINK-N-S-LIC and pFMgcLINK-N-H-LIC, respectively. Moreover, pFMgcLINK-N-SH-LINK was digested by *Mlu* I and *Pvu* II and ligated with the FLAG tag sequence composed of 2 oligonucleotides annealed to each other (Pvu-FLAG-Mlu-F: 5′ CTGGACTACAAGGATGACGATGACAAGA 3′, Pvu-FLAG-Mlu-R: 5′ CGCGTCTTGTCATCGTCATCCTTGTAGTCCAG 3′). This FLAG tag sequence was phosphorylated by T4 DNA kinase before its ligation. The next step was to construct pFMgcLINK-N-F-LIC. Human E1, E2, and E3 genes, which encode E1, E2, and E3 subunits of human KGDH, were amplified by PCR. Amplified E1, E2, and E3 genes lacked their mitochondrial targeting signal-coding sequences at their N-terminus (E1:1–41 a.a., E2: 1–67 a.a., E3: 1–43 a.a.). The primers used were as follows: (E1-F: 5′ TACTTCCAATCCAATGCACCTGTTGCTGCTGAGCCC 3′, E1-R: 5′ TCCACTTCCAATGCTACGAGAAGTTCTTGAAGAC 3′, E2-F: 5′ TACTTCCAATCCAATGATGACTTGGTTACAGTCAAAACC 3′, E2-R: 5′ TCCACTTCCAATGCTAAAGATCCAGGAGGAGGAC 3′, E3-F: 5′ TACTTCCAATCCAATGCAGATCAGCCGATTGATGCTG 3′, E3-R: 5′ TCCACTTCCAATGTCAAAAGTTGATTGATTTGCC 3′). Each amplified gene was inserted into pFMgcLINK-N-S-LIC, pFMgcLINK-N-H-LIC, and pFMgcLINK-N-F-LIC, respectively, using the LIC reaction. Then, pFMgcLINK-S-E1, pFMgcLINK-H-E2, and pFMgcLINK-F-E3 were constructed.

### Construction and extraction of recombinant BmNPV bacmids and injection into silkworm larvae or pupae

The construction and extraction of recombinant BmNPV bacmids were performed as described in a previous report [13]. *E. coli* BmDH10Bac CP^-^ strain was transformed with each pFastgcLINK vector or pFMgcLINK vector containing the gene of interest. Recombinant BmNPV bacmid harboring the gene of interest was extracted from white transformants. In this study, BmNPV-CP^-^-hMTP-EGFP, BmNPV-CP^-^-hMTP-S/hPDI-H, BmNPV-CP^-^-S-E1, BmNPV-CP^-^-H-E2, and BmNPV-CP^-^-F-E3 bacmids were constructed. Recombinant BmNPV bacmid DNA (∼10 µg) containing helper plasmid was mixed with a one-tenth volume of DMRIE-C (Invitrogen) reagent and this mixture was injected into silkworm larvae or pupae.

### Expression and purification of expressed proteins from fat bodies of silkworm larvae or pupae injected with recombinant BmNPV bacmid DNA

#### hMTP-hPDI complex

The fat bodies collected from 10 silkworm pupae were suspended in 25 mL of phosphate-buffered saline (PBS, pH 7.4) and sonicated 3 times for 30 sec each time with 1 min intervals. After centrifugation of the resultant homogenate at 8000×g for 10 min, the collected supernatant was mixed with 0.5 mL of PBS-equilibrated TALON Metal Affinity Resin (Clontech, Mountain View, CA, USA) and stirred at 4°C for 1 h. This mixture was centrifuged at 3000×g for 2 min, and the precipitated resin was washed with 120 mL of PBS. Proteins bound to resin were eluted with 2 mL of PBS containing 200 mM imidazole. This eluent was mixed and gently stirred with 0.25 mL of PBS-equilibrated Streptactin Superflow (QIAGEN K.K., Tokyo, Japan) at 4°C for 1 h. This mixture was centrifuged at 3000×g for 2 min and the supernatant was removed. After washing the affinity gel with 5 mL of PBS, hMTP-hPDI complex was eluted with 2 mL of PBS containing 5 mM desthiobiotin.

#### S-E1

The homogenate supernatant was obtained from the collected fat bodies of 5 silkworm larvae by the same manner as the purification of hMTP-hPDI complex. Supernatant was mixed with 0.5 mL of Strep-Tactin Superflow equilibrated with Tris-buffered saline (TBS, pH 7.5) and gently stirred at 4°C for 1 h. This mixture was centrifuged at 3000×g for 2 min and the supernatant was removed. After washing the affinity gel with 120 mL of TBS, S-E1 was eluted with 2 mL of TBS containing 5 mM desthiobiotin.

#### H-E2

H-E2: Homogenate supernatant was obtained from fat bodies collected from 5 silkworm larvae by the same manner as the purification of hMTP-hPDI complex. Supernatant was mixed with 0.5 mL of TALON Metal Affinity Resin equilibrated with TBS and gently shaken at 4°C for 1 h. This mixture was centrifuged at 3000×g for 2 min and the supernatant was removed. After washing the affinity gel with 120 mL of TBS, H-E2 was eluted with 2 mL of TBS containing 200 mM imidazole.

#### F-E3

Homogenate supernatant was obtained from fat bodies collected from 5 silkworm larvae by the same manner as the purification of hMTP-hPDI complex. Supernatant was mixed with 0.5 mL of anti-FLAG M2 antibody agarose (Sigma Aldrich Japan, Tokyo). This mixture was centrifuged at 3000×g for 2 min and the supernatant was removed. After washing the affinity gel with 120 mL of TBS, F-E3 was eluted with 2 mL of TBS containing 100 µg/mL of FLAG peptide (Sigma Aldrich Japan).

### SDS-PAGE and protein analysis

Recombinant protein samples were subjected to SDS-PAGE on 10 or 12% polyacrylamide gels using the Mini-protean II system (Bio-Rad Co. Ltd, Hercules, CA, USA). Total proteins on SDS-PAGE gel were detected with Coomassie Brilliant Blue (CBB) R-250 or silver staining. For the specific detection of fluorescent hMTP-EGFP fusion protein on SDS-PAGE gels, samples were mixed only with sample buffer and not boiled [14]. Fluorescent bands were detected using Molecular Imager FX (Bio-Rad). Fluorescent fusion proteins that were not boiled showed a slightly different molecular mass on SDS-PAGE gels as compared to boiled samples.

Protein concentration was determined using Protein Assay Kit II (Bio-Rad) based on the Bradford method.

Dialysis was performed in PBS overnight at 4°C using a dialysis membrane (WAKO Pure Chem. Ind., Ltd. Osaka, Japan).

Dihydrolipoamide dehydrogenase (DLD) activity was assayed according to Huo et al [15]. In brief, 1 mL of assay mixture [0.5 mM lipoamide, 0.1 mM NADH, 1 mM EDTA, 50 mM potassium phosphate (pH 6.7)] was incubated at room temperature, and 10 µL of enzyme was added to initiate the enzyme reaction. The decrease of absorbance at 340 nm was measured, and enzyme activity was calculated using the extinction coefficient of 6.22 mM^−1^ cm^−1^ for NADH. One unit of enzyme activity was defined as the amount of enzyme capable of catalyzing the conversion of 1 µmol of NADH to NAD^+^ per minute.

## Results and Discussion

### Expression of hMTP-EGFP fusion protein using pFast-SH-LIC vector

The hMTP-hPDI complex was expressed in silkworm larvae using newly constructed LIC vectors. hMTP is located in the lumen of endoplasmic reticulum (ER) as a heterodimer with human protein disulfide isomerase (hPDI) [16], [17]. This heterodimer catalyzes the transfer of triacylglycerol cholesteryl ester to phospholipid, and is involved in the assembly and secretion of very-low-density lipoprotein and chylomicrons. Bovine MTP was co-purified with bovine PDI from bovine liver homogenate [18]. Moreover, the hMTP was precipitated when expressed solely in insect cells, but it was solubilized and purified by only co-expression with hPDI [19].

**Figure 4:**
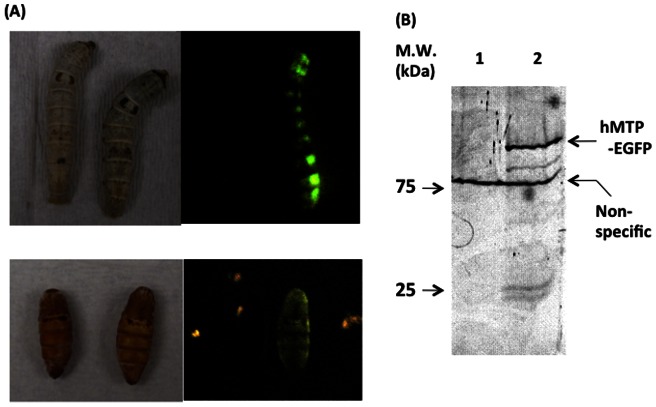
Expression of hMTP-EGFP fusion protein in silkworm larvae and EGFP fluorescence analysis of hMTP-EGFP fusion protein. (A) Expression of hMTP-EGFP fusion protein in silkworm larvae and pupae. (B) EGFP fluorescence analysis of hMTP-EGFP fusion protein in homogenate of silkworm larvae fat body on a SDS-PAGE gel. Lane 1: fat body of mock, lane 2: fat body of larvae into which bacmid DNA harboring hMTP-EGFP fusion gene was injected.

pFastBac-EGFP-SH-LIC contains the EGFP gene, Strep-tag II, and 6× histidine tag sequences downstream of the LIC region (*Sma* I site). EGFP gene, Strep-tag II, and 6× histidine tag sequences can be removed by thrombin due to the presence of a thrombin cleavage site between the LIC site and these tag sequences. The hMTP gene could be inserted with high efficiency at the LIC site by using the LIC reaction (identified 5 colonies that all had the hMTP gene-inserted vector). The LIC reaction is highly specific and the efficiency of its reaction is ∼100% [1], [2], [9]. Moreover, the hMTP-EGFP-SH gene sequence was confirmed to be correct in this plasmid. To confirm that hMTP-EGFP-SH fusion protein could be expressed in-frame using this vector, a recombinant BmNPV-CP^-^-hMTP-EGFP-SH bacmid was prepared and injected into silkworms and pupae. Green fluorescence was detected in silkworm larvae and pupae (Figure 4A). Fat bodies collected from larvae showing green fluorescence were suspended with PBS and sonicated to extract the expressed hMTP-EGFP fusion protein. A specific GFP fluorescent band on SDS-PAGE was observed in the homogenate of the bacmid-injected larvae's fat body compared to that of a mock injected fat body (Figure 4B). A non-specific fluorescent band was also observed in both homogenates, indicating that this band was from proteins inherent in silkworm larvae. These results indicate that the pFastBac-EGFP-SH vector works well and allows the expression of EGFP fusion protein.

### Construction of the co-expression vector of hMTP and hPDI

Co-expression vectors have been improved in the baculovirus expression system [20], [21], also in silkworms [22], but a digestion process using restriction enzymes is absolutely needed for cloning the genes of interest. In this study, a co-expression vector that does not require an enzymatic ligation reaction for gene cloning was constructed.

hMTP and hPDI genes were inserted using the LIC reaction into constructed pFastgcLINK-S-LIC and pFastgcLINK-H-LIC, respectively, (Figure 1) and then pFastgcLINK-hMTP-S and pFastgcLINK-hPDI-H were constructed as shown in Figure 2A. The efficiency of inserting hMTP and hPDI genes into pFastBac-S-LIC and pFastBac-H-LIC, respectively, was ∼100%. Sequences of hMTP connected with Strep-tag II (hMTP-S) and hPDI connected with 6× histidine tag (hPDI-H) were confirmed without any insertion and deletion of nucleotides (Figure 2C, only hMTP-S sequence is shown). pFastgcLINK-hMTP-S and pFastgcLINK-hPDI-H were connected by the LIC reaction as shown in Figure 2A. Normal and small sized transformants appeared after *E. coli* transformation of the LIC reaction. pFastgcLINK-hMTP-S/hPDI-H, which harbors hMTP-S and hPDI-H genes, was obtained from small sized transformants. The pFastgcLINK-hMTP-S/hPDI-H was obtained from all 4 small transformants, while pFastgcLINK-hMTP-S was obtained from all 4 normal transformants. The ratio of small transformants in all transformants was ∼50%; however, pFastgcLINK-hMTP-S/hPDI-H was obtained from small transformants with 100% efficiency. A recombinant BmNPV bacmid for co-expression of hMTP-S and hPDI-H was constructed using pFastgcLINK-hMTP-S/hPDI-H.

### Co-expression of hMTP-hPDI complex in silkworm larvae and pupae using constructed co-expression vector

**Figure 5:**
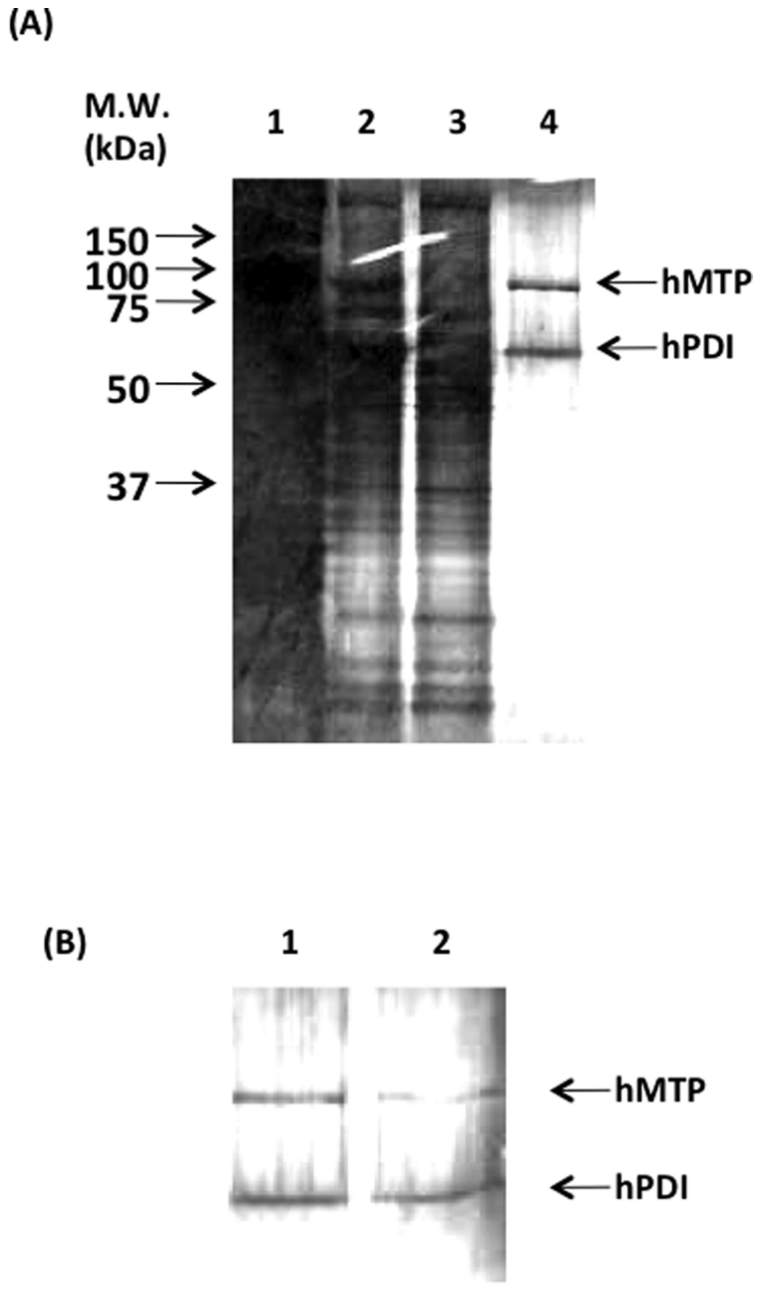
Analysis of purified hMTP-hPDI complex protein. The purified hMTP-hPDI complex protein was analyzed by SDS-PAGE with silver staining (A). Lane 1: homogenate of fat body, lane 2: eluent of TALON affinity chromatography, lane 3: flow through fraction of Strep-Tactin agarose chromatography, lane 4: eluent of Strep-Tactin agarose chromatography. (B) Comparison of purified hMTP-hPDI complex concentration in Strep-Tactin agarose chromatography. Lane 1: without dialysis before Strep-Tactin agarose chromatography, lane 2: with dialysis before Strep-Tactin agarose chromatography.

A recombinant BmNPV-CP^-^-hMTP-S/hPDI-H bacmid harboring hMTP-S and hPDI-H genes was injected to 10 pupae and silkworm larvae and reared for 6 d. The collected bacmid-injected pupae were suspended with PBS and homogenized by sonication. hMTP-hPDI complex was purified by TALON affinity resin and Strep-Tactin agarose because hMTP and hPDI were connected with Strep-tag II and 6× histidine tag, respectively. Two protein bands appeared after Strep-Tactin agarose chromatography (Figure 5A). The molecular weight of each band (hMTP-S and hPDI-H) was estimated to be ∼100 and 58 kDa, respectively, from each amino acid sequence. These values corresponded to 2 bands observed on a SDS-PAGE gel stained with silver staining. Moreover, no bands were observed in the Strep-Tactin agarose eluent except for hMTP-S and hPDI-H (Figure 5A). This indicates that hMTP-hPDI complex could be highly purified by TALON affinity resin and Strep-Tactin agarose. hMTP forms inclusion bodies when solely expressed in the baculovirus expression system [19], but hMTP formed the soluble complex with hPDI in pupae by co-expression with hPDI. This phenomenon is similar to a previous report on improvement of IgG solubility by co-expression of BiP in *Trichoplusia ni* cells using a baculovirus expression system [23]. Thirteen micrograms of purified hMTP-hPDI complex was obtained from 10 silkworm pupae. This value is less than that of human prorenin-human prorenin receptor complex (70 µg/15 silkworm larvae) reported previously [24]. However, the hMTP-hPDI complex from hemolymph of BmNPV-CP^-^-hMTP-S/hPDI-H bacmid DNA-injected silkworm larvae couldn't be purified. This suggests that hMTP-hPDI complex resides only in the endoplasmic reticulum (ER), and therefore, the yield of purified hMTP-hPDI complex was low compared to the yield of human prorenin-human prorenin receptor complex. To improve the recovery of purified hMTP-hPDI complex, purification by Strep-Tactin agarose was performed after dialysis of the hMTP-hPDI complex eluent of TALON affinity chromatography against PBS. Recovery yield (10 µg/10 silkworm pupae) was constant irrespective of dialysis (Figure 5B). When high-throughput purification of many recombinant proteins is performed in parallel using 2 purification steps, dialysis after the first chromatography is the rate-determining step. This affinity chromatography combination (6× histidine and Strep-tag II tags) is very useful for high-throughput protein purification because the dialysis step between TALON affinity and Strep-Tactin agarose chromatography can be omitted.

### Expression and purification of each human KGDH subunit in silkworm larvae using LIC vectors constructed for N-terminal tagged fused protein expression

Human KGDH is in the mitochondrial matrix and is a multi-enzyme complex in the TCA cycle. KGDH is composed of multi-copies of E1 (α-ketoglutarate dehydrogenase), E2 (dihydrolipoamide succinyltransferase), and E3 (dihydrolipoamide dehydrogenase). KGDH plays an important role in controlling the reductive potential (NADH/NAD^+^) in mitochondria [25]. Moreover, KGDH is a rate-limiting enzyme in the TCA cycle and may be involved in neurodegenerative disease [26]. Thus, expression of each KGDH subunit was investigated in silkworm larvae using LIC vectors without its mitochondrial targeting signal.

**Figure 6:**
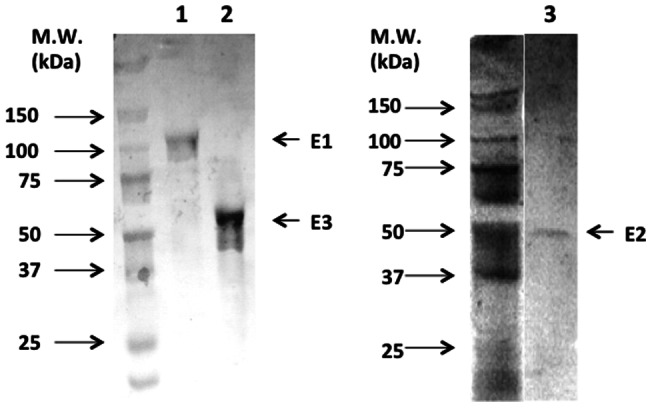
SDS-PAGE analysis of each purified KGDH subunit. The purified E1, E2, and E3 proteins were analyzed by SDS-PAGE with silver staining. Lane 1: E1 purified by Strep-Tactin agarose gel, lane 2: E3 purified by anti-FLAG M2 antibody agarose gel, lane 3: E2 purified by TALON affinity gel.

LIC vectors for the expression of recombinant proteins fused with an affinity tag at the N-terminus were also constructed as shown in Figure 3. The LIC cassette was initially amplified by PCR. The amplified fragment has a start codon, 6× histidine tag sequence, Strep-tag II tag sequence, TEV protease cleavage site sequence, and a LIC site. This LIC site sequence has the *Ssp* I site. However, pFastbac 1, which is based on all constructed LIC vectors in this study, also has a 4 *Ssp* I site. Then, 4 *Ssp* I sites in the constructed pFastgcLINK-N-SH-LIC vector, except for the *Ssp*I site in the LIC site, were mutated so as not to be digested by *Ssp* I. LIC vectors for each N-terminally tagged (6× histidine tag, Strep-tag II) protein, pFMgcLINK-N-S-LIC and pFMgcLINK-N-H-LIC, were constructed in the same manner as shown in Figure 1. Moreover, the FLAG tag sequence was replaced with a 6× histidine tag and Strep-tag II module at the *Mlu* I and *Pvu* II sites, and pFMgcLINK-N-F-LIC was constructed, indicating that any tag can be inserted into this site instead of the 6× histidine tag and Strep-tag II. This replacement can be available to LIC vectors constructed in Figure 1 for C-terminal tagged protein expression, or the pFastgcLINK-S-LIC or pFastgcLINK-H-LIC vector. To confirm the integrity of these vectors, each human KGDH subunit (E1, E2, E3) was inserted into pFMgcLINK-N-S-LIC, pFMgcLINK-N-H-LIC, and pFMgcLINK-N-F-LIC, respectively, using the LIC reaction. The efficiency of inserting the human KGDH subunit (E1, E2, E3) into pFastgcLINK-S-LIC or pFastgcLINK-H-LIC vectors was ∼100%, and each gene was inserted into each vector in-frame (Figure 3C, only E1 sequence is shown). Each recombinant bacmid was constructed using these vectors, and each subunit was expressed in silkworm larvae and purified from its fat body. Because each subunit lacked its mitochondria targeting signal-coding sequence, each subunit was purified from the supernatant of homogenate by affinity gel chromatography as almost a single band at an estimated molecular weight (E1: 110 kDa, E2: 48 kDa, E3: 55 kDa); except that some amount of purified E3 was degraded and had slightly higher mobility on an SDS-PAGE gel (Figure 6.). E3 is a DLD and DLD activity was assayed using purified E3. E3 was purified from the fat body homogenate by ∼1224-fold, and the specific activity of purified E3 was 3.2 U/mg (Table 1). In this study, 55, 30, 130 µg of purified E1, E2, and E3 were obtained from 5 bacmid-injected silkworm larvae, respectively. This verified that LIC vectors pFMgcLINK-N-S-LIC, pFMgcLINK-N-H-LIC, and pFMgcLINK-N-F-LIC also work well for protein expression and purification in silkworms.

**Table 1:** Purification of dihydrolipoamide dehydrogenase expressed in fat body of silkworm larvae.

	Volume (ml)	DLD activity (U)	Protein concentration (mg)	Specific activity (U/mg)	Purification (-fold)*	Recovery (%)
Fat body homogenate	20	0.32	121.00	2.64×10^−3^	1	100
Anti-FLAG agarose	2	0.42	0.13	3.23	1224	130

* Defined by dividing specific activity by that of fat body homogenate.

## Conclusions

LIC and co-expression vectors were constructed using a new gcLINK sequence and BmNPV bacmid. Using these vectors, an hMTP-hPDI co-expression vector was successfully constructed without ligation. Moreover, purification of hMTP-hPDI complex was easily performed by TALON affinity and Strep-Tactin agarose chromatography, because Strep-tag II and 6× histidine tag were fused with hMTP and hPDI, respectively. Moreover, LIC vectors for the expression of recombinant proteins fused with several tags in the N- or C-terminal end were constructed. Each human KGDH subunit was expressed in silkworm larvae and purified from its fat body. This indicates that LIC vectors constructed in this study were available for protein expression and purification in silkworms. Because these vectors facilitate high-efficiency cloning of genes and fusion with purification tags, they are applicable for high-throughput expression of proteins, co-expression of protein complexes, and simultaneous administration of gene expression in the silkworm expression system.
